# HDAC4 Is Indispensable for Reduced Slow Myosin Expression at the Early Stage of Hindlimb Unloading in Rat Soleus Muscle

**DOI:** 10.3390/ph14111167

**Published:** 2021-11-16

**Authors:** Inna I. Paramonova, Natalia A. Vilchinskaya, Boris S. Shenkman

**Affiliations:** Myology Laboratory, Institute of Biomedical Problems RAS, 123007 Moscow, Russia; vilchinskayanatalia@gmail.com (N.A.V.); bshenkman@mail.ru (B.S.S.)

**Keywords:** hindlimb unloading, Tasqiunimod, MyHC, HDAC4, soleus muscle

## Abstract

It is well known that reduced contractile activity of the main postural soleus muscle during long-term bedrest, immobilization, hindlimb unloading, and space flight leads to increased expression of fast isoforms and decreased expression of the slow isoform of myosin heavy chain (MyHC). The signaling cascade such as HDAC4/MEF2-D pathway is well-known to take part in regulating MyHC I gene expression. Earlier, we found a significant increase of HDAC4 in myonuclei due to AMPK dephosphorylation during 24 h of hindlimb unloading via hindlimb suspension (HU) and it had a significant impact on the expression of MyHC isoforms in rat soleus causing a decrease in MyHC I(β) pre-mRNA and mRNA expression as well as MyHC IIa mRNA expression. We hypothesized that dephosphorylated HDAC4 translocates into the nuclei and can lead to a reduced expression of slow MyHC. To test this hypothesis, Wistar rats were treated with HDAC4 inhibitor (Tasquinimod) for 7 days before HU as well as during 24 h of HU. We discovered that Tasquinimod treatment prevented a decrease in pre-mRNA expression of MyHC I. Furthermore, 24 h of hindlimb suspension resulted in HDAC4 nuclear accumulation of rat soleus but Tasquinimod pretreatment prevented this accumulation. The results of the study indicate that HDAC4 after 24 h of HU had a significant impact on the precursor MyHC I mRNA expression in rat soleus.

## 1. Introduction

Skeletal muscle consists of different fiber types and have different functions. Slow-type fibers are fatigue resistant, focused on prolonged contraction duration and postural control, but have reduced maximum contraction force and velocity. Fast-type fibers provide high contraction force and velocity but fatigue quickly. It has been discovered that these features of muscle fibers are determined by myosin phenotype, i.e., expression of slow and fast isoforms of myosin heavy chain (MyHC). Gravitational unloading in space flight and simulated microgravity on Earth lead to the myosin phenotype transformation most in the postural soleus muscle—m. soleus. It is well known that reduced contractile activity of the main postural soleus muscle during long-term bedrest, immobilization, hindlimb unloading, and space flight leads to increased expression of fast isoforms and decreased expression of the slow isoform of myosin heavy chain (MyHC) [1,2,3,4,5]. It has been shown that a seven-day spaceflight led to a slow-to-fast shift in the fiber type ratio in soleus rat muscles [6]. Additionally, resistive exercises during bed rest prevented myosin phenotype transformation [7]. For the first time, it was shown that a decrease in the content of MyHC Iβ mRNA occurs already on the 4th day in unloaded soleus muscle of rat [2]. However, a significant decline in precursor MyHC I mRNA expression and mature MyHC I mRNA expression in rat soleus muscle has been observed as early as on the 1st day of hindlimb unloading via hindlimb suspension (HU) [5,8]. This decline could be associated with the selective activity of two main signaling pathways: HDAC4/MEF2-D pathway and calcineurin/NFATc1. In active muscle fiber, calcineurin dephosphorylates NFATc1 and promotes its translocation into the myonuclei. In the nuclei, NFATc1 directly interacts with MEF2 transcription factors that specifically bind the slow-type MyHC gene promoter and activate its expression [9,10]. Thus, intense slow-type MyHC transcription is triggered. Under muscle unloading, NFATc1 content in the nuclei significantly decreased in rat soleus muscle as early as on the 1 day of hindlimb unloading [11]. The mechanisms of this decrease still remain unclear. Possibly, this decrease may be caused by the observed GSK-3beta (Ser9) phosphorylation level decrease in soleus muscle after the first day of hindlimb unloading [11]. The signaling cascade HDAC4/MEF2-D pathway is well-known to take part in regulating MyHC I gene expression [5,9,10]. As previously shown, HDAC4 mediates gene repression by the recruitment to MEF2 sites in the promoters of repressed genes [12]. DNA-bound MEF2 transcription factors through interaction with class IIa HDACs would recruit the HDAC activity to deacetylate local chromatin and repress transcription. It has been found that class IIa HDACs act as common transcriptional repressors of many promoters which are controlled by MEF2 transcription factors [13]. The ability of class IIa HDACs to act as potent inhibitors of MEF2-dependent transcription is widely documented [14,15,16,17,18,19].

In addition to HDAC4, the transcriptional activity of MEF2 is controlled by various repressors, including muscle-specific repressors such as myogenic regulatory factor 4 (MRF4). MRF4 appears to exert its repressive effect on MEF2 via a multiprotein repressive complex containing HDAC4 and the NCoR1 corepressor, as shown by the discovery that MRF4 knockdown induces nuclear export of HDAC4 [20]. In recent years, it has been shown that MRF4 acts as a negative regulator of muscle growth by suppressing MEF2 [21]. It should be noted that HDAC4 first of all deacetylates nucleosomal histones. There are very few data on histone acetylation under hindlimb unloading; however, it has been shown that HU induced an increase in histone H3 acetylation at the type IIb (fast) MyHC and deacetylation of histones H3 at the type I (slow) MyHC [22]. Protein acetylation is also regulated by a number of different HATs, for example, histone acetyltransferase p300, since this nuclear cofactor is involved in the regulation of muscle cell differentiation [23] and muscle atrophy [24,25,26,27]. p300 regulates the acetylation of both histones and non-histone proteins, including transcription factors and nuclear cofactors involved in the regulation of muscle mass, such as NF-kB/p65 [28], FOXO transcription factors [29], C/EBPβ [30], and PGC-1α [31].

HDAC4 activity is controlled by two main mechanisms: through altered nuclear cytoplasmic traffic by phosphorylation and the formation of complexes with other proteins. Phosphorylation of class IIa HDACs, including HDAC4, leads to the dissociation of the protein complex with transcription factors and the translocation of class IIa HDACs into the cytoplasm [32]. Being dephosphorylated HDAC4, shuttles to the nuclei and interact with transcription factors and histones to block *myh7* (slow-type MyHC) gene activity [33,34]. Therefore, phosphorylation of HDAC4 prevents its import into the myonuclei [10,34,35]. HDAC4 can be phosphorylated by calcium/calmodulin-dependent protein kinase II (CaMKII), protein kinase D (PKD) and AMP-activated protein kinase (AMPK) [34,35,36,37,38].

Earlier, we found a significant increase of HDAC4 in myonuclei due to AMPK dephosphorylation during 24 h of hindlimb unloading via hindlimb suspension (HU) and it had a significant impact on the expression of MyHC isoforms in rat soleus causing a decrease in MyHC I(β) pre-mRNA and mRNA expression as well as MyHC IIa mRNA expression [5]. It remains unknown whether HDAC4 increase in the nuclei may mediate a decrease in slow MyHC expression. We hypothesized that dephosphorylated HDAC4 translocates into the nuclei and can lead to a reduced expression of slow MyHC. To test this hypothesis, Wistar rats were treated with HDAC4 inhibitor (Tasquinimod) for 7 days before HU as well as during 24 h of HU. We examined whether nuclear content alteration and activity of HDAC4 facilitate slow MyHC mRNA expression shift. Previous studies used Tasquinimod to inhibit HDAC 4 using rat models [39], mice models [40,41], and cell lines [42]. The mechanism of tasquinimod action is through targeting the histone acetylation of genes through blocking HDAC4. It was shown that Tasquinimod binds to HDAC4 in the zinc-binding regulatory domain to lock the protein in a conformation preventing HDAC4/N-CoR/HDAC3 complex formation which lead to inhibiting deacetylation of histones and HDAC4 target transcription factors [42]. Results were also obtained showing that 3 days of unloading with inhibition of HDAC4/5 by trichostatin also affected the nuclear content of HDAC4 in rat soleus muscle [43]. Therefore, it is possible that the mechanism of inhibition of HDAC 4 includes inhibition of its traffic to the nucleus.

## 2. Results

**Figure 1:**
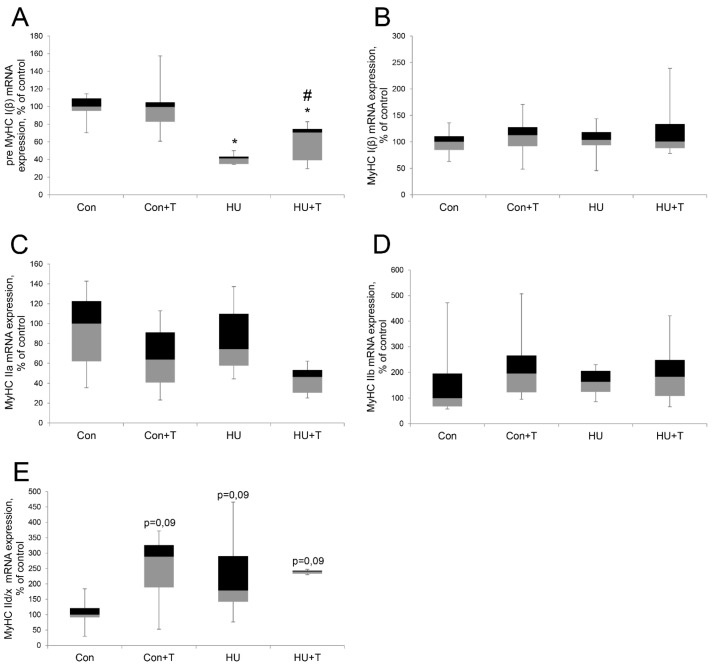
Expression levels of pre *MyHC I(β)* (**A**)*, MyHC I(β)* (**B**), *MyHC IIa* (**C**), *MyHC IIb* (**D**), *MyHC IId/x* (**E**), and mRNAs in rat soleus muscle in the control group (Con), control group with Tasquinimod treatment (Con + T), 24 h of hindlimb unloading via hindlimb suspension (HU), 24 h of hindlimb unloading via hindlimb suspension with Tasquinimod treatment (HU + T). Data are shown as % of the control group. *—significant difference from the control group. #—significant difference from HU group (*p* < 0.05). Box plots show 25–75 percentiles and median values and the whiskers represent the minimum and the maximum; *n* = 8/group.

We studied the expression of slow and fast MyHC isoforms (Figure 1A–E). After 24 h of hindlimb unloading via hindlimb suspension, Precursor of type I myosin mRNA transcription was significantly lower relative to the control group and in the Tasquinimod treatment group (HU + T) the level of precursor type I myosin mRNA transcription was significantly decreased also relative to the control group, but significantly increased compared the HU group (*p* < 0.05). However, mature type I myosin mRNA transcription did not differ among the groups. Fast-type myosin IIA and IIB mRNAs both did not differ among the groups. Fast-type myosin IId/x mRNA had a tendency to an increase compared the control group (*p* = 0.06) (Figure 1A–E).

**Figure 2:**
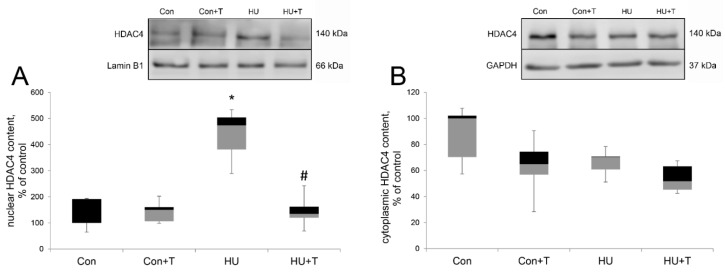
Western blot analysis of nuclear HDAC4 (**A**) and cytoplasmic HDAC4 (**B**) content in rat soleus muscle in control group (Con), control group with Tasquinimod treatment (Con + T), 24 h of hindlimb unloading via hindlimb suspension (HU), 24 h of hindlimb unloading via hindlimb suspension with Tasquinimod treatment (HU + T). Data are shown as % of the control group. *—significant difference from the control group. #—significant difference from HU group (*p* < 0.05). Box plots show 25–75 percentiles and median values and the whiskers represent the minimum and the maximum; *n* = 8/group.

After 24 h of hindlimb unloading via hindlimb suspension, the nuclear content of HDAC4 (343%) was more than three-fold above the control (*p* < 0.05). In the Tasquinimod treatment group (HU + T) the level of HDAC4 was significantly reduced compared the HU group (Figure 2A). The cytoplasmic content of HDAC4 did not change among the groups (Figure 2B).

**Figure 3:**
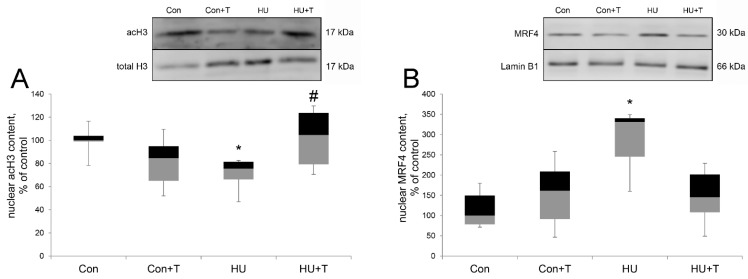
Western blot analysis of acH3 (**A**) and MRF4 (**B**) nuclear content in rat soleus muscle in control group (Con), control group with Tasquinimod treatment (Con + T), 24 h of hindlimb unloading via hindlimb suspension (HU), 24 h of hindlimb unloading via hindlimb suspension with Tasquinimod treatment (HU + T). Data are shown as % of the control group. *—significant difference from the control group. #—significant difference from HU group (*p* < 0.05). Box plots show 25–75 percentiles and median values and the whiskers represent the minimum and the maximum; *n* = 8/group.

Histones H3 is a substrate for HDAC4. The nuclear content of acetylated H3 was significantly reduced in the HU group compared to the control group (*p* < 0.05) and in the Tasquinimod treatment group (HU + T) the level of acetylated H3 was significantly increased compared the HU group. (Figure 3A). The nuclear content of MRF4 (231%) during 24 h of hindlimb unloading (HU) was significantly increased compared to the control group (*p* < 0.05). In the Tasquinimod treatment group (HU + T) the level of MRF4 was the same as in the Con group (Figure 3B).

**Figure 4:**
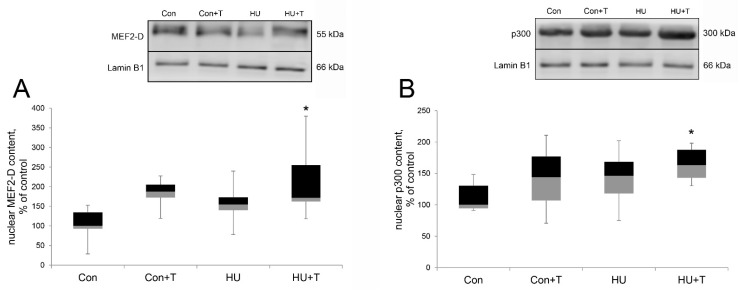
Western blot analysis of MEF2-D (**A**) and p300 (**B**) nuclear content in rat soleus muscle in control group (Con), control group with Tasquinimod treatment (Con + T), 24 h of hindlimb unloading via hindlimb suspension (HU), 24 h of hindlimb unloading via hindlimb suspension with Tasquinimod treatment (HU + T). Data are shown as % of the control group. *—significant difference from the control group (*p* < 0.05). Box plots show 25–75 percentiles and median values and the whiskers represent the minimum and the maximum; *n* = 8/group.

After 24 h of hindlimb unloading via hindlimb suspension, the nuclear content of MEF2-D and p300 did not differ from the control group; however, in the Tasquinimod treatment group (HU + T) the levels of MEF2-D and p300 were significantly increased compared to the control group (*p* < 0.05) (Figure 4A,B).

**Figure 5:**
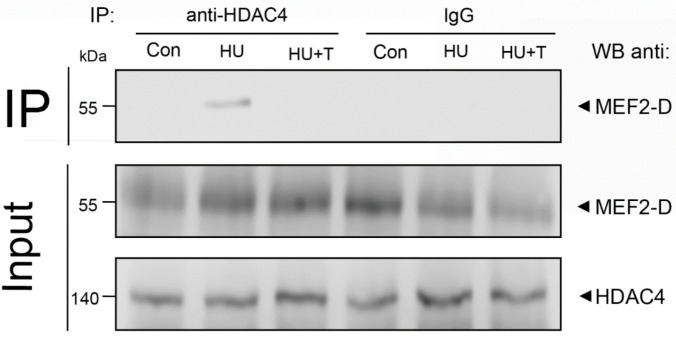
Co-immunoprecipitation of the HDAC4 with the MEF2-D.

We also performed co-immunoprecipitation of HDAC4 with MEF2-D in the muscle lysate of the rat soleus muscles. We found that after 24 h of hindlimb unloading via hindlimb suspension, HDAC4 binds directly to MEF2-D, forming a complex, but this complex was not detected in the control group or in the group with Tasquinimod treatment (Figure 5).

Muscle lysates were immunoprecipitated (IP) with polyclonal antibodies against the HDAC4, and then probed by Western blot (WB) with antibodies against MEF2-D. A positive control (input) confirmed the presence of MEF2-D in the muscle lysate prior to immunoprecipitation. Nonspecific IgG immunoprecipitation for each experimental group was used as a negative control. The panel is a representative Western blot from the experiment.

Input—positive control, Con—control group, IgG Con—negative control for the Control group, HU—24 h of hindlimb unloading via hindlimb suspension, IgG HU—negative control for HU group, HU + T—24 h of hindlimb unloading via hindlimb suspension with Tasquinimod treatment, IgG HU + T—negative control for the HU + T group.

## 3. Discussion

Earlier, we found a significant increase of HDAC4 in myonuclei due to AMPK dephosphorylation during 24 h of hindlimb unloading via hindlimb suspension (HU) and it had a significant impact on the expression of MyHC isoforms in rat soleus causing a decrease in MyHC I(β) pre-mRNA and mRNA expression as well as MyHC IIa mRNA expression [5]. We hypothesized that dephosphorylated HDAC4 translocates into the nuclei and can lead to a reduced expression of slow MyHC. It remains unknown whether HDAC4 abundance increase in the nuclei may mediate a decrease in slow MyHC expression. To test this hypothesis, Wistar rats were treated with HDAC4 inhibitor (Tasquinimod) for 7 days before HU as well as during 24 h of HU. Previous studies used Tasquinimod to inhibit HDAC 4 using rat models [39], mice models [40,41], and cell lines [42]. Results were also obtained showing that, after 3 days of unloading with inhibition of HDAC4/5 by trichostatin, the nuclear content of HDAC4 in rat soleus muscle [43] was also affected. Therefore, it is possible that the mechanism of inhibition of HDAC4 includes not only inhibition of its deacetylase activity, but inhibition of its traffic to the nucleus.

We studied the slow and fast isoforms of MyHC expression. Precursor of slow myosin mRNA transcription significantly decreased after 24 h of hindlimb suspension. These data are in good agreement with the results obtained under similar conditions on Sprague-Dawley animals [8], as well as with our previous data obtained after 24 h of hindlimb suspension [5]. Tasquinimod treatment also resulted to Precursor slow myosin mRNA transcription decrease during unloading, but less pronounced than in hindlimb suspension. Precursor slow myosin mRNA transcription significantly increased in the Tasquinimod hindlimb suspension group compared to hindlimb suspension group. Thus, partial prevention of precursor slow myosin mRNA transcription decrease was associated with Tasquinimod treatment. We did not find significant differences of mature slow myosin mRNA transcription in all experimental groups. Tasquinimod treatment during hindlimb suspension had no effect on mature slow myosin mRNA transcription. Apparently, the time of action of hindlimb unloading in our experiment had effect only on precursor slow myosin mRNA transcription and had not yet affected the mature slow myosin mRNA transcription. The data obtained confirm our assumptions about the role of HDAC4 in the regulation of immature slow myosin mRNA transcription and correlates with the data on the nuclear-cytoplasmic traffic of HDAC4. No differences in the fast IIA myosin mRNAs transcription were found; however, we previously noted the fast IIA myosin mRNAs transcription decreases after 24 h of hindlimb suspension [5]. We did not find significant differences in fast IIB myosin mRNAs transcription in all groups. These data are consistent with the results in the experiment using AICAR, where fast IIB myosin mRNAs transcription also did not change [5]. We found a tendency to the fast IId/x myosin mRNAs transcription increase after 24 h of hindlimb suspension; it is interesting to note that Tasquinimod treatment led to an increase of the fast IId/x myosin mRNAs transcription also during unloading, but more pronounced than in the group of hindlimb suspension. In this context, it is possible that HDAC4 is also involved in the stabilization of the “fast” myosin phenotype due to increased expression of fast myosin isoforms under hindlimb unloading.

We examined whether the activity of HDAC4 facilitate slow MyHC mRNA expression shift. We found a significant increase of HDAC4 nuclear content relative to the control group after 24 h of hindlimb suspension. This result is in good agreement with our earlier data on AMPK-dependent accumulation of HDAC4 in the nuclei in rat m. soleus after 24 h of hindlimb suspension [5] and the work of Yoshihara et al., who showed HDAC4 increase in the nuclei in rat m. gastrocnemius after 10 days of immobilization [44]. Treatment with Tasquinimod during unloading returns HDAC4 nuclear content to the control level, which allows us to conclude that the HDAC4 inhibitor Tasquinimod blocked its nuclear content increase while HDAC4 cytoplasmic content in rat soleus muscles did not have significant differences between the groups. It should be noted that inhibition of HDAC4 primarily affected the nuclear content of HDAC4—it is reduced. Similar results were obtained with HDAC1 inhibition by CI-994 [45] and with HDAC4 inhibition by trichostatin [43]. Therefore, it is possible that the mechanism of inhibition of histone deacetylases includes inhibition of its traffic to the nucleus in skeletal muscle. Furthermore, HDACs play a critical role in the repression of gene transcription by histone deacetylation and increasing chromatin condensation [12,14]. We evaluated the acetylation levels of the N-terminal end of histone H3 in order to evaluate the deacetylase activity of HDAC4 in rat m. soleus after 24 h of hindlimb suspension. Previously, a deep decrease was found in acetylated histone linked with the myh7(slow MyHC) gene promoter after 7 days of HU [22]. HDAC4 deacetylates histone H3, and indeed, Tasquinimod treatment prevented unloading-induced histone H3 acetylation decrease, and one of the causes for this alteration may be HDAC4 nuclear content change. However, HDAC4 deacetylates not only histone H3, but also the MEF2-D transcription factor, which controls the promoter activity of the myh7 gene. Histone deacetylase 4 can accumulate in the nuclei of muscle cells and suppress the expression of various genes by directly binding and inhibiting the activity of the transcription factor MEF2 [5,14,35,44]. After 24 h of hindlimb suspension the MEF2-D nuclear content did not differ from the control, which is consistent with our earlier data on this time point [46]. However, we found that Tasquinimod treatment during unloading led to a significant increase of the MEF2-D nuclear content in rat soleus muscle. It is not yet clear what is the cause for this increase. It is possible that MEF2-D is a non-canonical target for other kinases. Additionally, it is possible that the return of the MRF4 nuclear content to the control level in the Tasquinimod group activates the transcriptional activity of MEF2-D and leads to the subsequent activation of muscle-specific genes, which are known to be targets for MEF2 [20].

We also performed co-immunoprecipitation of HDAC4 with MEF2-D in the muscle lysate of the rat soleus muscles. We found that after 24 h of hindlimb suspension, HDAC4 binds directly to MEF2-D, forming a complex, and this complex was not detected in the control group and in the group with the Tasquinimod treatment. The data obtained confirm our hypothesis about direct binding of HDAC4 to MEF2-D, which leads to deacetylation and inhibition of the transcriptional activity of MEF2-D, which controls the promoter activity of the myh7 gene after 24 h of hindlimb suspension in the rat soleus muscle. However, we do not eliminate the possibility that there is one or more intermediate molecules involved in binding of these two molecules that were assayed. In the Tasquinimod group, HDAC4 does not bind to MEF2-D, possibly due to the observed decreased nuclear content of HDAC4 in this group. In addition to histone deacetylase 4, the transcriptional activity of MEF2 is controlled by various repressors, including muscle-specific repressor such as myogenic regulatory factor 4 (MRF4) and nuclear receptor corepressor 1 (NCoR1). MRF4 appears to exert its repressive effect on MEF2 via a multiprotein repressive complex containing HDAC4 and the NCoR1 corepressor, as shown by the discovery that MRF4 knockdown induces nuclear export of HDAC4 [20]. In our experiment we found that the nuclear content of MRF4 was significantly increased after 24 h of hindlimb suspension; however, in the group with Tasquinimod treatment during unloading, this difference was not found. We hypothesize that this effect is associated with the nuclear HDAC4 abundance increase in hindlimb suspension group, while the histone deacetylase 4 inhibitor-Tasquinimod blocks nuclear content increase of MRF4 and HDAC4 also (together with HDAC4). Probably, HDAC4 together with MRF4 enter the myonuclei. However, the molecular mechanisms of this import are still unknown and need further study. Additionally, it has been shown that HDAC4 is involved in MRF4-dependent repression of MEF2, since it has been shown that the accumulation of HDAC4 in the nucleus caused by denervation is markedly reduced by the Mrf4 knockdown [20]. The activity of not only histone deacetylases, but also histone acetyltransferases affects the expression of muscle genes. Studies of p300 activity in skeletal muscles under functional unloading have not been carried out. Nevertheless, it is known that activation of the MyHC type I promoter is realized by p300, which in turn acetylates NFATc1, which facilitates its binding to the promoter [47]. We found a significant increase of the p300 nuclear content relative to the control level after 24 h of hindlimb suspension with Tasquinimod treatment. However, there was no increase in the p300 nuclear content in hindlimb suspension group. Probably, histone deacetylase 4 counteracts the nuclear accumulation of histone p300 acetyltransferases, which are necessary for normal muscle gene expression [23]. Several studies showed that HATs and HDACs provide a link between the signal pathways that regulate muscle cell differentiation and various transcription factors that activate muscle genes directly [23]. Additionally, hypo-acetylated histones are associated with transcriptionally silent genes, consistent with the fact that the stimulatory effects of HATs on gene expression are counteracted by HDACs [23]. However, the molecular mechanisms of p300 import into muscle nuclei are unknown and require further study.

## 4. Materials and Methods

### 4.1. Ethical Approval

All procedures with the animals were reviewed and approved by the Biomedicine Ethics Committee of the Institute of Biomedical Problems of the Russian Academy of Sciences/Physiology Section of the Russian Bioethics Committee (protocol no. 2, 28.05.2021). All experiments were performed in strict accordance with the Guiding Principles of American Physiological Society in the Care and Use of Vertebrate Animals in Research and Training. All animals were kept in a temperature-controlled room on a 12:12-h light-dark cycle with food pellets and water provided ad libitum. Wistar male rats were acquired from the certified nursery for laboratory animals of the Institute of Bioorganic Chemistry of the Russian Academy of Sciences (Pushchino, Moscow region). Prior to all surgical procedures, the animals were euthanized by intraperitoneal injection of a tribromoethanol overdose (750 mg/kg) followed by cervical dislocation. The anesthesia depth was evaluated by testing the pedal withdrawal reflex (toe and foot pad pinch).

### 4.2. Study Design

Male Wistar rats weighing 180–225 g (three-month-old) were randomly divided into four groups (8 animals in each): control group (Con), control group with the administration of a HDAC4 inhibitor (Tasquinimod) at a concentration of 10 mg/kg body weight per day orally (Con + T), hindlimb suspended group for 24 h (HU), hindlimb suspended group for 24 h with the administration of a HDAC4 inhibitor (Tasquinimod) at a concentration of 10 mg/kg body weight per day orally (HU + T). Control and HU groups of animals received a placebo equivalent in volume. Previous studies used Tasquinimod to inhibit HDAC4 using Wistar rat models at a concentration 10 mg/kg/day with food since this optimal dose had effect on HDAC 4 and acetylation of histones [39]. «HU + T» group of Wistar rats were treated with Tasquinimod (#A3860, ApexBio, Houston, TX, USA) at 10 mg/kg/day (administered orally with a small amount of food) for 7 days before HU as well as during 24 h of HU. The same conditions were used for the «Con + T» group of Wistar rats, but without HU.

On completion of the experiment, the rats were euthanized as described above, and their soleus muscles were rapidly removed and immediately frozen in liquid nitrogen until later analysis. The animals from the control groups were euthanized on the same day as the HU and HU + T groups.

### 4.3. Hindlimb Suspension Protocol

The animals were subjected to gravitational unloading (hindlimb unloading) conditions using a standard hindlimb suspension model [48,49]. A detailed description of the hindlimb suspension protocol can be found in our previous reports [50,51]. This model causes atrophy of the postural muscles.

### 4.4. Protein Extraction and Western Blot Analysis

A detailed description of protein extraction and Western blotting procedures can be found in our previous report [5].

In brief, muscle samples were loaded and separated on a 10% polyacrylamide gel, followed by transfer to a nitrocellulose membrane (Santa Cruz Biotechnology, Inc., Sanford, ME, USA, #sc-3724), after which membranes were incubated in a blocking buffer (TBS-T: 4% non-fat milk powder; Tris-buffered saline, pH 7.4; and 0.1% Tween 20). The membranes were then incubated with primary and secondary antibodies and washed in TBS-T. The primary antibodies used were GAPDH (1:10,000, Applied Biological Materials Inc., Richmond, BC, Canada, # G041), Lamin B1 (1:500, Abcam, Cambridge, MA, USA, # ab16048), MEF2-D (1:1000, EMD Millipore, Temecula, CA, USA, # AB2263), acetyl-Histone H3 (1:1000, EMD Millipore, Temecula, CA, USA, # 06-599), total Histone H3 (1:1000, Cell Signaling Technology, Danvers, MA, USA, # 9715), HDAC4 (1:500, Cell Signaling, Danvers, MA, USA, #2072), HAT P300 (1:500, Abcam, Cambridge, MA, USA, # ab231010).

Secondary HRP-conjugated antibodies (1:30,000) to rabbit or mouse immunoglobulins were from Santa Cruz Biotechnology, CA, USA. Protein bands were detected and quantified using Clarity Western ECL Substrate (Bio-Rad Laboratories, Hercules, CA, USA, #170-5061) and C-DiGit Blot Scanner (LI-COR Biotechnology, Lincoln, NE, USA).

### 4.5. Co-Immunoprecipitation

Co-immunoprecipitation was prepared from 40 mg of frozen soleus muscle samples using The Thermo Scientific Pierce Co-Immunoprecipitation Kit (Thermo Fisher Scientific, Waltham, MA, USA) according to the manufacturer’s protocol. Muscles were solubilized in lysis buffer (0.025M Tris, 0.15M NaCl, 0.001M EDTA, 1% NP-40.5% glycerol; pH 7.4) with Complete Protease Inhibitor Cocktail (Santa-Cruz), Phosphatase Inhibitor Cocktail B (Santa Cruz), PMSF (1 mM), aprotinin (10 μg/mL), leupeptin (10 μg/mL), and pepstatin A (10 μg/mL). Immunoprecipitation was carried out using rabbit polyclonal antibody against the HDAC4 (Abcam, # 12172). After incubation with coupling resin for overnight at 4 °C, the immunocomplex was washed three times in lysis buffer. The protein samples were heated for 5 min at 95 °C in loading buffer, run on 10% separating SDS-polyacrylamide gel, and probed with the primary polyclonal antibodies against the MEF2-D (1:1000, EMD Millipore, Temecula, CA, USA, # AB2263). The secondary VeriBlot for IP Detection Reagent HRP-conjugated antibodies (1:1000, Abcam, Cambridge, MA, USA, # ab131366) were used for a 1-h incubation at room temperature. Then the blot was revealed using the ImmunStar TM Substrate Kit (Bio-Rad Laboratories, USA) and the C-DiGit Blot Scanner (LI-COR Biotechnology, Lincoln, NE, USA). Muscle lysate prior to immunoprecipitation was used as a positive control (input). Muscle lysates with non specific rabbit IgG (Santa Cruz, CA, USA, #2027), for each experimental group were used as negative controls.

### 4.6. RNA Analysis

RT-PCR analysis was performed as reported previously [5,51]. Briefly, total RNA extraction was provided using the RNeasy Micro Kit according to the manufacturer’s recommendations (Qiagen, Hilden, Germany). 0.5 μg RNA was reverse-transcribed to cDNA using the RevertAid RT Kit (Thermo Scientific) according to the manufacturer’s instruction.

The compared samples were processed under similar conditions (template amounts, duration of PCR cycles). Real-time amplification was monitored using SYBR Green I and the iQ5 multicolor real-time PCR detection system (Bio-Rad Laboratories, USA). PCR primers used for RNA analysis are shown in Table 1. RPL19 was used as the housekeeping gene. The Pfaffl method was used to calculate of relative gene expression.

### 4.7. Statistical Analysis

All PCR and Western blot data are expressed as median and interquartile range (0.25–0.75) of eight animals. The median values of all groups are shown as a percentage of the control group. Statistical analysis was provided using the REST 2009 v.2.0.12 (Qiagen, Germany) and Origin Pro v.8.0 (OriginLab Corp., Northampton, MA, USA) programs. Given the small sample sizes and comparisons among four groups, significant differences between groups were statistically analyzed using Kruskal-Wallis nonparametric test followed by Dunn’s post hoc test. Differences with values of *p* <0.05 were regarded as statistically significant.

## 5. Conclusions

In conclusion, the current study showed that after 24 h of hindlimb unloading Tasquinimod treatment prevented a decrease in pre-mRNA expression of MyHC I. Twenty-four hours of hindlimb suspension resulted in HDAC4 accumulation in the nuclei of rat soleus but Tasquinimod treatment prevented this accumulation. The results of the study indicate that HDAC4 after 24 h of HU had a significant impact on the precursor MyHC I mRNA expression in rat soleus.

## Figures and Tables

**Table 1:** Primers used for RT-PCR analysis.

Gene Description	Forward Primer Reverse Primer	GenBank
*pre myh7 (MyHC I(β))*	5′-ACTTAGCAGGCAAATCTCAGTAGC-3′ 5′-CTCGCGTTATGTTTCTCATCCGAAT-3′	NM_017240.2
*Myh7* (MyHC I(β))	5′-ACAGAGGAAGACAGGAAGAACCTAC-3′ 5′-GGGCTTCACAGGCATCCTTAG-3′	NM_017240.2
*RPL19*	5’-GTACCCTTCCTCTTCCCTATGC-3’ 5’-CAATGCCAACTCTCGTCAACAG-3’	NM_031103.1
*Myh2 (MyHC IIa)*	5′-TATCCTCAGGCTTCAAGATTTG-3′ 5′-TAAATAGAATCACATGGGGACA-3′	NM_001135157.1
*Myh4 (MyHC IIb)*	5′-CTGAGGAACAATCCAACGTC-3′ 5′-TTGTGTGATTTCTTCTGTCACCT-3′	NM_019325.1
*Myh1 (MyHC IId/x)*	5′-CGCGAGGTTCACACCAAA-3′ 5′-TCCCAAAGTCGTAAGTACAAAATGG-3′	NM_001135158.1

## Data Availability

Data is contained within the article.
